# Supplementary material for: Transcriptome sequencing and screening of genes related to the MADS-box gene family in Clematis courtoisii
Source: PLoS One. 2024 Feb 5;19(2):e0294426. doi: 10.1371/journal.pone.0294426 (PMC10843124; doi:10.1371/journal.pone.0294426)
Supplement: S1 Data — (ZIP) [file pone.0294426.s001.zip › Basic_data/readme.docx]

Readme

Folder: data_analysis

T01.subreads.stat.xls: subreads length, reads number statistics, average length, N30, N50, N90, base number statistics

T01.flnc_stat.xls: Assembly sequence information statistics

Transcript.fasta: consensus sequence

Unigenes.fasta: gene sequences after clustering

Unigenes.100.ANGEL.cds: cds sequence analysis

Unigenes.100.ANGEL.pep: protein sequence analysis

Unigenes.100.ANGEL.utr: utr sequence analysis

Folder:Unigene_Anno(unigene annotation result):

Integrated_Function.annotation.xls: Annotation result statistics for all genes.

Unigenes.fasta.Cog_class.txt: COG annotation results for all genes.

Unigenes. Fasta. GO. Anno.txt: of all genes GO annotation results.

Unigenes.fasta.Kog_class.txt: KOG annotation results for all genes.

Unigenes. Fasta. Nr. Anno.txt: all nr gene annotations.

Unigenes. Fasta. Pfam. Anno.txt: all genes Pfam annotation results.

Unigenes. Fasta. Swissprot. Anno.txt: all genes Swissprot annotation results.

CcMADS and Homologous protein.fasta: Related protein sequence information

newick tree.txt: A phylogenetic tree file
