# Supplementary material for: Transcriptome sequencing and screening of genes related to the MADS-box gene family in Clematis courtoisii
Source: PLoS One. 2024 Feb 5;19(2):e0294426. doi: 10.1371/journal.pone.0294426 (PMC10843124; doi:10.1371/journal.pone.0294426)
Supplement: Attachment — Submitted filename: Response to Reviewers.docx [file pone.0294426.s002.docx]

**Response to editor’s and reviewers’ Comments**

**Reviewer 1:**

Concerns about the clarity and depth of the introduction, particularly regarding the MADS-box gene family identification.
Questions about the replicates used in full-length transcriptome sequencing.
The method for identification and analysis of MADS-box genes was seen as not rigorous enough.
Clarity is needed in the methods and parameters for multi-sequence alignment and phylogenetic tree construction.
The figures provided are blurry and need to be clearer and more visually appealing.
The narrative lacks logical progression, and the relationship between transcriptome sequencing and gene family identification is not clear.
Reviewer suggests a more in-depth analysis, emphasizing the need for a deeper dive into the gene family identification.

**Reply:** Thank you very much for your constructive comments. We have taken your kind suggestions.

1. L67-L70 The last paragraph of the introduction should include the MADS-box gene family identification.

We have added the related introduction about MADS-box gene family identification in the L80-88 of the tracked version manuscript.

2. L75 Just use a clematis? Are there no replicates set up in full-length transcriptome sequencing?

The project was initiated with the objective of investigating the impact of three generations of sequencing in order to identify the genes associated with flower development in C. courtoisii. For sequencing, only C. courtoisii was chosen as the subject of research, and samples from different developmental stages were collected, ground, and mixed to serve as the material. We have added the related introduction in the L96-97 of the tracked version manuscript.

3. L114 The method of identification and analysis of MADS-box genes is not rigorous, usually in addition to using homologous genes for blatsp, it is necessary to identify the members of the gene family through the unique domain of the family, and check the integrity of the domain, and the family's conservative motif is not analyzed later, so I am skeptical of the results.

The genes related to the mads-box were initially screened based on the annotation results from the database in the L150-167 of the tracked version manuscript. Subsequently, these sequences were uploaded to the SMART online database for verification of their sequence structures, and further compared and validated using hmmer V3.0 software's hummscan function with MADS-box conserved domain pfm files PF00319 and PF01486. Ultimately, a total of 19 candidate MADS-box-associated genes were identified. Additional steps for analyzing conserved motif alignment with model plants are provided in the L180-187 of the tracked version manuscript.

4. L123-L124 Please add the methods and parameters for multisequence alignment and phylogenetic tree construction.

The method and parameter settings for multiple sequence alignment and phylogenetic tree construction using MEGA software are supplemented in the L169-178 of the tracked version manuscript.

5. L147 “CDS is a protein coding sequence that is completely consistent with the codon of the protein”, which is confusing.

We have revised the article by eliminating this section.

6. L225 Please clarify the basis for the selection of the 97 MADS-box related genes.

We modified the screening method and utilized the hmmscan function of the hmmer software to compare translated protein sequences of Unigenes against downloaded hmm files SRF-TF (PF00319) and K-box (PF01486), thereby identifying a total of nineteen directly associated MADS-box Unigenes. It should be noted that only MADS-box-related genes identified based on annotated information were used as reference.

7. L267 There does not appear to be a comparison of second- and third-generation transcriptomes in the manuscript.

The transcriptome sequencing of C. courtoisii was limited to a single third-generation analysis, lacking both a reference genome and a reference second-generation transcriptome. Consequently, the second-generation sequencing data from other clematis species were utilized for comparison and analysis.

8. All the pictures are blurry and can't see the legend clearly. The figures seem not attractive enough, especially figure 7 and 5, maybe the author should reorganize the complex information in a visually appealing way.

The requirements of the figure were carefully reviewed and necessary modifications were made to ensure the uploaded pictures met high quality standards.

**Reviewer 2:**
Concerns about the quality of figures and the necessity for high-quality uploads.
A lack of detail in the methodology, especially concerning de novo transcript assembly.
Questions about the significance of identifying lncRNAs.
Concerns about the functional annotation approach and its implications.
The need for a deeper interpretation and discussion regarding the phylogenetic analysis results.

**Reply:** Thank you very much for your constructive comments. We have taken your kind suggestions.

1. The figures are of extremely poor quality and I am unable to read or interpret anything. Please upload high quality figures otherwise reviewing this manuscript properly will be impossible

The requirements of the figure were carefully reviewed and necessary modifications were made to ensure the uploaded pictures met high quality standards.

2. The authors mention that they do not use a genome or transcriptome reference but provide no detail on how they perform de novo transcript assembly. Detailed methods on how these transcripts were identified is important to assess the novelty of their results

We have added the parameters and Settings of the software used in the process to L102-131 of the tracking version of the manuscript.

3. They identify lncRNAs as well as protein coding genes but do not provide reasons for why this may or may not be important

We have revised the article by eliminating this section.

4. The authors use hits against databases such as Pfam, KEGG etc. as a proxy for "functional annotation". They do not mention the background sets of the genes used which can severely affect the results obtained, and additionally do not provide any interpretation of function. This in my opinion is extremely misguiding to a reader.

The quality of transcriptome sequencing data is crucial for accurate gene function annotation. High-quality data can enhance the reliability and precision of gene function annotation, thereby facilitating a better understanding of gene functions in organisms. However, the method used to screen C. courtoisii MADS-box related genes from gene functional annotations lacks rigor. In order to assess the transcriptome quality, we utilized the mentioned gene functional annotations (accounting for.19% of transcripts) as an indicator. To further search and identify MADS-box related genes, we modified our screening approach by employing hm V3.0 software's hummscan function to compare and validate conserved domain files PF00319 and PF01486 associated with MADS-box proteins. As a result, nineteen Unigenes directly linked to MADS-box were successfully identified.

5. The authors perform phylogenetic analysis to identify MADS-box genes. Once again, they do not comment on the novelty or implications of their findings and their figure legends as well as methods are lacking with regard to interpretability.

we have made further modifications to the figure format in order to present a well-defined phylogenetic tree and facilitate comparison of conserved motifs. This will enhance our understanding of the type, quantity, and function of MADS-box genes associated with flower formation and development in C. courtoisii.

**Here's what we recommend for the revision:**
Revisit the introduction, ensuring clarity and depth, particularly regarding the MADS-box gene family identification.
Provide clarity on the replicates used in full-length transcriptome sequencing.
Detail your methodology, especially concerning de novo transcript assembly and the identification and analysis of MADS-box genes.
Address the concerns raised about the figures and ensure they are of high quality and easily interpretable.
Revisit the narrative to ensure logical progression and clear relationships between different sections.
Dive deeper into the gene family identification, providing a comprehensive and in-depth analysis.
Address all other specific concerns raised by the reviewers.

The revision of the references has been meticulously carried out in provided by the reviewers. We sincerely appreciate your reminder.
